# A BioBrick™-Compatible Vector for Allelic Replacement Using the *XylE* Gene as Selection Marker

**DOI:** 10.1186/s12575-016-0036-z

**Published:** 2016-02-13

**Authors:** Michela Casanova, Lorenzo Pasotti, Susanna Zucca, Nicolò Politi, Ilaria Massaiu, Cinzia Calvio, Maria Gabriella Cusella De Angelis, Paolo Magni

**Affiliations:** Department of Electrical, Computer and Biomedical Engineering, Laboratory of Bioinformatics, Mathematical Modelling and Synthetic Biology, University of Pavia, 27100 Pavia, Italy; Centre for Health Technologies, University of Pavia, 27100 Pavia, Italy; Department of Biology and Biotechnology, University of Pavia, 27100 Pavia, Italy

**Keywords:** Allelic replacement, BioBrick, Knockout, Standard vector, *XylE*

## Abstract

**Background:**

Circular plasmid-mediated homologous recombination is commonly used for marker-less allelic replacement, exploiting the endogenous recombination machinery of the host. Common limitations of existing methods include high false positive rates due to mutations in counter-selection genes, and limited applicability to specific strains or growth media. Finally, solutions compatible with physical standards, such as the BioBrick™, are not currently available, although they proved to be successful in the design of other replicative or integrative plasmids.

**Findings:**

We illustrate pBBknock, a novel BioBrick™-compatible vector for allelic replacement in *Escherichia coli*. It includes a temperature-sensitive replication origin and enables marker-less genome engineering via two homologous recombination events. Chloramphenicol resistance allows positive selection of clones after the first event, whereas a colorimetric assay based on the *xylE* gene provides a simple way to screen clones in which the second recombination event occurs. Here we successfully use pBBknock to delete the lactate dehydrogenase gene in *E. coli* W, a popular host used in metabolic engineering.

**Conclusions:**

Compared with other plasmid-based solutions, pBBknock has a broader application range, not being limited to specific strains or media. We expect that pBBknock will represent a versatile solution both for practitioners, also among the iGEM competition teams, and for research laboratories that use BioBrick™-based assembly procedures.

**Electronic supplementary material:**

The online version of this article (doi:10.1186/s12575-016-0036-z) contains supplementary material, which is available to authorized users.

## Background

A large number of methods, recently reviewed by Song et al. [1], are available for the efficient genome engineering of *Escherichia coli* and other bacteria. Among them, circular plasmid-mediated homologous recombination is commonly used for marker-less allelic replacement, exploiting the endogenous recombination machinery of the host. In such method, a mutated version of the target locus is cloned in a conditional-replication plasmid, together with the two DNA sequences flanking it. Upon transformation, a first cross-over event integrates the plasmid in the target chromosomal region and a second one excises the integrated plasmid, leaving the allele with the desired modifications without any plasmid DNA sequences. While clones in which the first cross-over successfully occurs are easily selected via antibiotic resistance, the second cross-over is a rare event and clones that have lost the plasmid are usually screened via a counter-selection method [1]. Finally, the counter-selected clones, which have the same theoretical probability (50 %) to contain the desired modified allele or to maintain the original state, need to be screened by PCR [2]. The counter-selection gene most widely used in this type of plasmids is *sacB*, which converts sucrose into a toxic product, thus enabling the selection of clones in growth media containing this sugar [3]. Apart from the requirement of specific media, a reported drawback of such popular method is the spontaneous mutation that can occur in *sacB*, resulting in false positive clones [4]. Other counter-selection methods available, such as those based on the *rpsL*, *galK*, *thyA*, *tetA* and *tolC* genes, also present strong strain and/or medium limitations [5, 6]. The I-*Sce*I counter-selection system has been proposed to overcome such issues [7], but false positive clones due to mutations can still occur at high frequency [8]. This is a common feature of synthetic kill switches implemented via toxic genes [9], although combination of multiple counter-selection systems has been reported to decrease the false positive rate [6]. Methods have been proposed that use temperature-sensitive vectors without toxic genes, exploiting the integrated replication origin to stimulate the second recombination event in permissive (replicative) conditions [10]. This strategy, coupled with a *lacZ* gene-mediated blue/white screening, is successfully used in Gram positive bacteria [11], although its use in *E. coli* would be limited to specific *lacZ*-mutant strains.

**Fig. 1:**
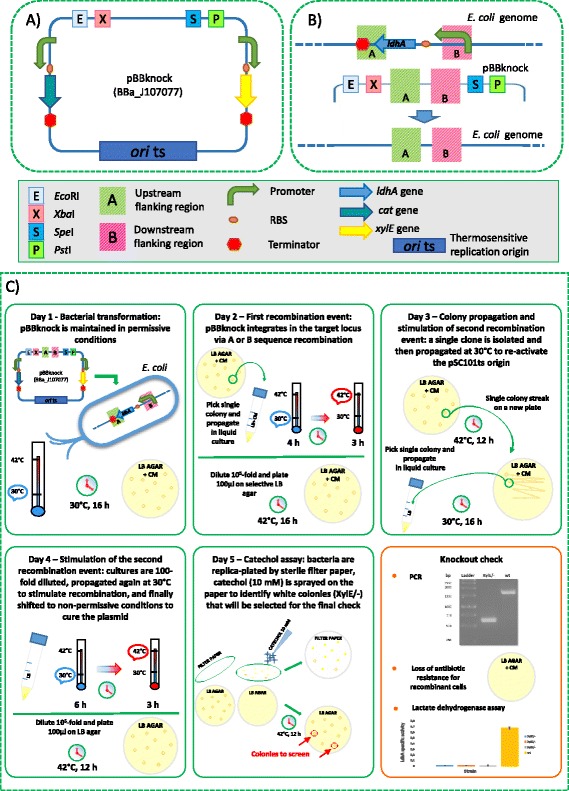
Description of the pBBknock vector, knockout experimental design and protocol. **a** Vector description; all the elements are described in the box below the panel. **b** The AB DNA sequence is assembled in the pBBknock vector and the resulting plasmid is used to carry out chromosomal gene deletion via two successive recombination events, described in panel **c**. After the two recombination events, the resulting genomic target sequence is shown: it has about 50 % probability to be successfully modified or to revert to the wild type state (not shown). **c** Allele replacement protocol description. Notes on protocol development are reported in Additional file 1: Notes on protocol development

In this work, we propose a new vector (pBBknock, see Fig. 1a) for allelic replacement in *E. coli* that exploits a temperature-sensitive replication origin and the *xylE* gene from *Pseudomonas putida*, coding for the catechol 2,3-dioxygenase enzyme [12]. This enzyme is not toxic for *E. coli* (data not shown) and converts the colourless substrate catechol into the yellow product 2-hydroxymuconic semialdehyde within seconds, resulting in a cheap and fast colorimetric assay to identify clones in which the second recombination event, i.e., plasmid excision, has not occurred. Although the *xylE* gene has previously been used as a reporter for gene expression in different microorganisms, such as *Bacillus subtilis*, *Actinosynnema pretiosum* and *Streptomyces* spp [12–14], its application as selection marker in marker-less genome engineering protocols for *E. coli* represents a novel aspect of this work. XylE is encoded by a single 0.9-kbp gene and its activity can be detected without the requirement of specific strains or media. It was preferred over other available reporter systems for coloured product formation because the latter have less attractive features for pBBknock: violacein and carotenoid pathways are encoded by large multi-genic constructs [15]; the single gene for melanin production requires specific medium formulation [16]. Finally, fluorescent reporters can be hard to detect when expressed from low or single DNA copies.

Since the development of standard genetic tools is one of the hallmarks of synthetic biology, strongly facilitating and speeding up the recombinant strain construction process [17, 18], we designed a vector that is compatible with commonly used BioBrick™ standards (RFC10, RFC12 and RFC23) [19]. This novel plasmid for allelic replacement represents an advanced genetic tool in the ready-to-use BioBrick™-compatible vectors for genome engineering that have been recently proposed by our group [20], which, although enabling marker-less genome engineering, still introduce plasmid-derived sequences surrounding the target locus.

## Results

The pBBknock vector includes a pSC101ts temperature-sensitive origin (BBa_J107112) derived from pAH123 [21] [GenBank: AY048726] (see Additional file 1: Details about pBBknock thermosensitive sequence design). The vector also carries a chloramphenicol resistance cassette (BBa_P1004) including the *cat* gene with its own promoter and ribosome binding site (RBS) and the *xylE* gene with its own RBS (BBa_J33204) under the control of the BBa_J23101 constitutive promoter [19]. BBa_J23101 is a medium-strength promoter that is widely used in synthetic biology studies and often serves as a standard reference in promoter characterization experiments [20, 22–24]. We used BBa_J23101 to drive the *xylE* expression in preliminary experiments in different strains and plasmid copy numbers and, according to catechol plate assay, the resulting expression cassette was functional and did not significantly reduce bacterial growth rate (data not shown). The L3S2P42 and L3S3P22 synthetic transcriptional terminators [25] are used downstream of the *cat* and *xylE* cassette, respectively. Properly-placed unique *Eco*RI, *Xba*I, *Spe*I and *Pst*I restriction sites constitute the BioBrick™-compatible cloning site. The vector was fully constructed via the GenScript (Piscataway, NJ, USA) gene synthesis service.

The design specifications described above, including heterologous and synthetic components, allowed us to obtain a BioBrick™-compatible vector with a significantly low level of similarity to the *E. coli* genome, thus minimizing the off-target integration probability. The pBBknock sequence (see Fig. 1a) can be accessed as BBa_J107077 in the Registry of Standard Biological Parts [19] and its DNA is available upon request.

As expected, the resulting vector replicates in *E. coli* at 30 °C and not at 42 °C. The copy number of pBBknock is very similar to the one of pSB4C5, demonstrating that in permissive conditions the pSC101ts origin is maintained at a copy number comparable with the one of a vector with the non-ts pSC101 low-copy number origin (see Additional file 1: Copy number characterization).

We used pBBknock to delete the lactate dehydrogenase (*ldhA*) gene in the chromosome of *E. coli* W, a widely used strain in metabolic engineering studies [26]. In particular, A and B sequences were designed, constructed and ligated to pBBknock to delete the chromosomal sequence comprised between the *ldhA* core promoter region (annotated in [EcoCyc: G592]) and the last 7 codons of the coding sequence (see Fig. 1b).

The process followed to achieve the gene knockout, inspired by Hamilton et al. [10] and Arnaud et al. [11], is described in Fig. 1c. Among 6 independent experiments, white colonies (i.e., with successful vector excision) ranged from 1 % to 11 % of the total colonies, with a 4 % mean occurrence. Ten white clones were screened by colony PCR: three of them were successful knockout strains, while the others maintained the original allele (see Fig. 1c). Gene deletion was also confirmed by the absence of lactate dehydrogenase activity in the three *ldhA*^-^ strains (see Fig. 1c).

## Discussion

This work develops a novel allelic replacement vector, merging physical standardization concepts and a screening procedure based on a simple colorimetric assay, never applied before in marker-less allelic replacement methods for *E. coli*, that can be virtually used with any growth medium and host. The false positive rate is expected to be lower than in counter-selection systems based on toxic genes, which can frequently mutate (see Additional file 1: Notes on protocol development). However, allelic replacement efficiency may vary in different strains and experiments, according to the host recombination capability, allele-dependent fitness, and flanking sequence length and homology [27]. Homologous sequences can be retrieved from a specific collection of BioBrick™ parts [20] or can be easily constructed via PCR (as it was carried out in this work). BioBrick™ parts can also be assembled between the two homologous DNA regions to be integrated in the target locus. Since pBBknock is replicated at low copy, it is particularly suited to deliver difficult parts (toxic when present in high copy) in the chromosome, for which other plasmid-based methods, e.g., the ones using the conditional R6K origin which is replicated at medium or high copy, may not be successful [5, 21]. Although novel promising techniques for large-scale genome editing have been developed [1], the modification of a single gene via the plasmid-based *sacB* method is still commonly carried out in many laboratories [28–30]. Efficient one-step methods based on linear DNA are also commonly used [1, 31], but they require a helper plasmid expressing specific recombinases and are applicable only to limited bacterial strains, since others might suffer from poor transformation efficiency with linear fragments.

In this view, we expect that pBBknock will represent a versatile solution both for practitioners, also among the iGEM competition teams, and for research laboratories that use BioBrick™-based assembly procedures.

## Materials and Methods

### *E. coli* Strains, Reagents and Cloning

TOP10 (Invitrogen) were used for cloning according to manufacturer's instructions. For gene knockout experiments, the W strain (ATCC 9637) was transformed by a standard heat shock protocol [32]. Strains were routinely grown in LB medium; chloramphenicol (12.5 mg/l) or ampicillin (100 mg/l) were added as required. Catechol (C9510, Sigma Aldrich) was dissolved in deionized water to obtain a 10 mM stock that was prepared fresh every day. Primers used in this work are listed in Additional file 1: Table S1.

The pBBknock vector was specialized to delete the *ldhA* gene of *E. coli* W by assembling the *ldhA* flanking DNA fragments (A and B, both 0.9 kbp-long, see Fig. 1b) in the cloning site. A and B regions were separately amplified from the genome of *E. coli* W with primer pairs PAtail_F/PAtail_R and PBtail_F/PBtail_R, respectively, with Phusion Hot Start Flex polymerase (New England Biolabs). Each PCR product was purified (NucleoSpin Extract II, Macherey-Nagel), digested with *Eco*RI and *Pst*I (Roche), purified again, and finally individually ligated (T4 ligase, Roche) into the *Eco*RI-*Pst*I-digested pSB1A2 vector [19]. Each construct was sequence-verified with standard BioBrick™ primers VF2 and VR. The A and B fragments in pSB1A2 were then digested with *Spe*I-*Pst*I and *Xba*I-*Pst*I, respectively, and ligated according to the BioBrick™ Standard Assembly to yield the AB construct (in pSB1A2), which was sequence-verified and, upon *Eco*RI-*Pst*I digestion, finally ligated into pBBknock.

### Lactate Dehydrogenase Assay

The assay was performed as described by Massaiu et al. [23]. Cultures grown to saturation at 37 °C at 220 rpm in 2 ml of LB with 100 mM phosphate buffer and 40 g/l glucose, were 100-fold diluted in 9 ml of the same medium and grown for 4 h. One ml of culture was centrifuged (13,000 rpm, 1 min), washed with 1 ml of 100 mM Tris-HCl pH 7.3 and the pellet was resuspended with 0.4 ml of CelLytic B (Sigma Aldrich), supplemented with a protease inhibitor cocktail, to lyse the cells. After 10 min at room temperature, cell debris were removed by centrifugation (13,000 rpm, 5 min) and the supernatant was assayed. Reaction mix (180 μl), containing 100 mM Tris-HCl pH 7.3, 0.4 mM NADH and 10 mM sodium pyruvate, was mixed with 20 μl of lysate and absorbance at 340 nm (OD_340_) was monitored at 25 °C every 5 min in an Infinite F200 (Tecan) microplate reader. The slope of the absorbance time series, proportional to enzymatic activity of the sample, was computed via linear regression. Protein quantification in the lysate was obtained via Micro BCA Protein Assay Kit (Thermo Scientific). Specific enzymatic activity was calculated by dividing the total enzymatic activity by protein level and expressed as 10^4^*OD_340_/min/μg of cell protein.

### Copy Number Estimation for pBBknock

The copy number of pBBknock was estimated by comparing it to the one of pSB4C5 [19], which carries a non-ts pSC101 origin. To this aim, the BBa_J107029 part containing a constitutive promoter driving the Red Fluorescent Protein (RFP) expression, was assembled in both vectors upon *Eco*RI-*Pst*I digestion. Transformed TOP10 cells were assayed both in selective LB and M9 supplemented medium (11.28 g/l M9 salts - M6030, Sigma Aldrich, 2 mM MgSO_4_, 0.1 mM CaCl_2_, 2 g/l casamino acids, 1 mM thiamine hydrochloride and 4 ml/l glycerol) as previously reported [20], except that cultures were always incubated at 30 °C. RFP synthesis rate per cell (S_cell_), expressed in arbitrary units (AU), was computed and assumed to be proportional to the plasmid copy number. S_cell_ and cell growth rate were computed as previously described [20]. Results were expressed as average S_cell_ values of at least three biological replicates and the confidence intervals of S_cell_ mean were reported.

## Additional File

Additional file 1: Supplementary notes, results, figures and tables.
**Figure S1.** Growth curves for TOP10 strain bearing pBBknock or a control vector (pSB4C5) with pSC101 replication origin. **Figure S2.** Probability of finding at least one illegal BioBrick™ restriction site in a nucleotide window of variable length in the genome of *E. coli W.* **Table S1.** Primers used in this study.(DOCX 70 kb)
