# Supplementary material for: A BioBrick™-Compatible Vector for Allelic Replacement Using the XylE Gene as Selection Marker
Source: Biol Proced Online. 2016 Feb 13;18:6. doi: 10.1186/s12575-016-0036-z (PMC4752771; doi:10.1186/s12575-016-0036-z)
Supplement: Additional file 1: Supplementary notes, results, figures and tables. — Figure S1. Growth curves for TOP10 strain bearing pBBknock or a control vector (pSB4C5) with pSC101 replication origin. Figure S2. Probability of finding at least one illegal BioBrick™ restriction site in a nucleotide window of variable length in the genome of E. coli W. Table S1. Primers used in this study.(DOCX 70 kb) [file 12575_2016_36_MOESM1_ESM.docx]

Supplementary information

**1 - Supplementary notes and results**

**1.1 - Details about pBBknock thermosensitive sequence design**

The pBBknock vector was obtained via de-novo synthesis. In order to design it, the sequence of the pSC101ts temperature-sensitive replication origin was retrieved and modified to remove unwanted restriction sites (*Spe*I). In this process we found inconsistencies among the available sequences for some widely used temperature-sensitive vectors, which are described below.

BBa_I50052 (pSC101ts) is a thermo-sensitive version of BBa_I50042 (pSC101), both present in the Registry; the mutation conferring the temperature-sensitive phenotype of BBa_I50052 is annotated. Consistently, this mutation is also found in the sequence of the temperature-sensitive pKOV vector [2] [https://www.addgene.org/25769]; conversely, in the deposited sequence of another widely used temperature-sensitive vector, pAH123 [21] [GenBank: AY048726], such mutation is not reported. Because of such inconsistency, we sequenced pAH123 with primers P1_F, P2_R, P3_F, P4_F, P5_R (reported in Table S1) and we could confirm the presence of the temperature-sensitive mutation described in pSC101ts and pKOV; thus, the sequence given in the [GenBank: AY048726] entry does not include the temperature-sensitive nucleotide change. Since comparison between our sequence of pAH123 and the one of BBa_I50052 showed additional mismatches (not shown), we decided to rely on pAH123 origin to design our vector. We extended the origin region to the upstream *Nco*I restriction site and to the downstream stop codon of the ampicillin resistance gene in pAH123. We also modified the origin region by removing the *Spe*I site, following the strategy used to modify pSC101 (BBa_I50042), present in the widely used pSB4C5 vector, to ensure that the nucleotide changes do not affect the replication origin functioning. The resulting sequence is annotated in the BBa_J107077 entry of the Registry as temperature-sensitive replication origin pSC101ts and has been submitted as part BBa_J107112.

**1.2 - Notes on protocol development**

The second recombination is a rare event and the corresponding step of the protocol (DAY 3-4, Figure 1C) is critical. No white colonies were present in catechol-stained plates if the incubation at 30°C was carried out for 6 hours only; an overnight incubation, followed by a 100-fold dilution and additional 6-hour incubation, was essential to obtain positive clones in the strain and conditions tested. Also, no white colonies were present in catechol-stained plates if the incubation temperature of DAY 3-4 (Figure 1C) was set at 42°C instead of 30°C, demonstrating that the second recombination event is stimulated only at permissive temperature for the pSC101ts origin. Since no white colonies were present in such conditions, these results also suggest that the false positive rate (i.e., the occurrence of white colonies in which the second recombination did not happen) is negligible in the host strain and condition tested.

**1.3 - Copy number characterization**

Measured fluorescence values (used to estimate plasmid copy number) were similar between pBBknock and pSB4C5: S_cell_=1.52±0.17 AU and 1.48±0.29 AU, respectively, in LB, and 3.34±0.03 AU and 2.65±0.05 AU, respectively, in M9. Doubling times of recombinant strains bearing pBBknock and pSB4C5 were 78 min and 54 min, respectively, in LB, and 161 min and 112 min, respectively, in M9. Figure S1 shows the growth curves of *E. coli* bearing pBBknock or pSB4C5.

**1.4 - Occurrence of illegal restriction sites in homologous fragments**

Here we report the probability of finding at least one illegal restriction site (*Eco*RI, *Xba*I, *Spe*I or *Pst*I) when designing a homologous fragment to be cloned, like A or B in this work. To perform this task, we considered the *Eco*RI, *Xba*I, *Spe*I or *Pst*I restriction sites in the ATCC 9637 genome sequence. By using a nucleotide window moving along the genome, we found via Perl script this probability, shown in Figure S2 as a function of the nucleotide window length. The considered lengths have been chosen in accordance with the length of homologous fragments used in previous studies [2,3,10]. Although a high probability of finding at least one site (about 26%) is present for a length of 0.9 Kb, i.e., the one used in this work, this probability dramatically decreases when a lower length is considered, e.g., about 16% for 0.5 Kb, successfully used in several applications [2].

**Figure S1.** Growth curves for TOP10 strain bearing pBBknock or a control vector (pSB4C5) with pSC101 replication origin. Both vectors have BBa_J107029 as insert. Data are relative to the copy number characterization experiment carried out in microplate reader [20]. Data represent background-subtracted absorbance (OD_600_) over time in cultures grown in LB or M9 supplemented medium. Solid lines represent the mean of four independent clones, while dotted lines are the 95% confidence intervals of the mean.


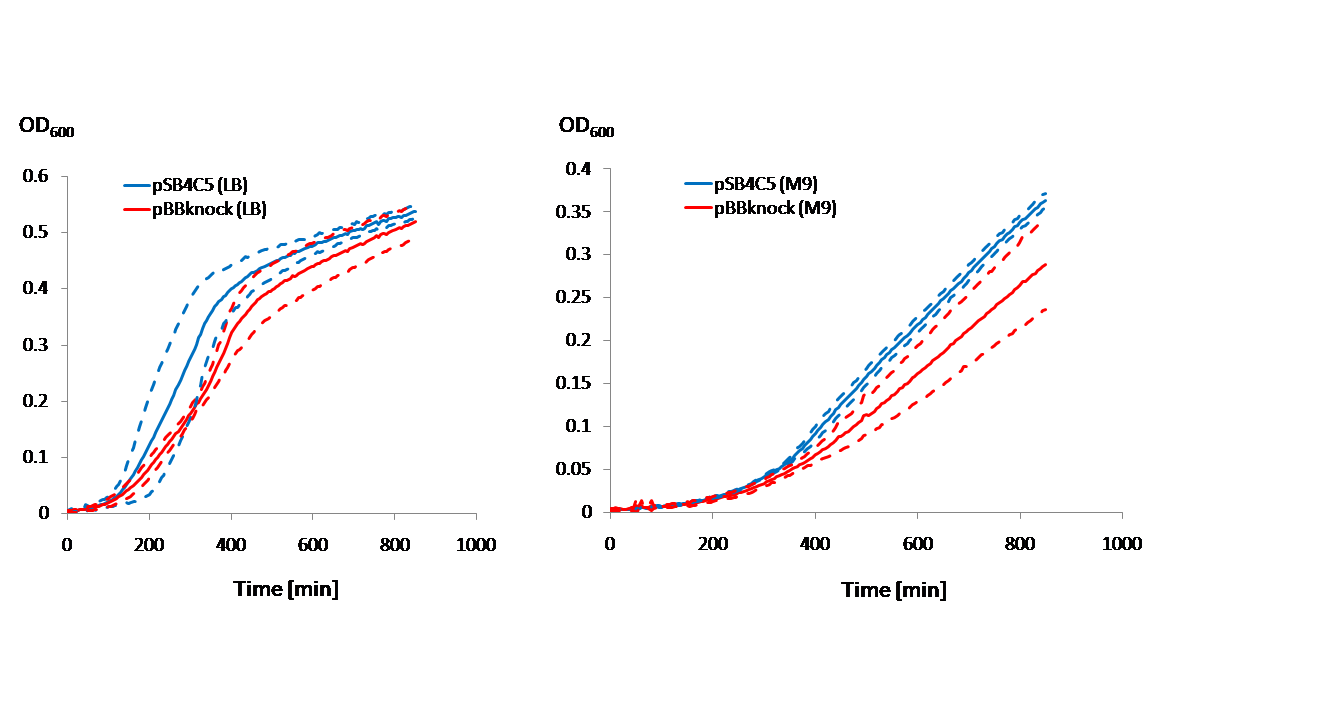


**Figure S2.** Probability of finding at least one illegal BioBrick^TM^ restriction site in a nucleotide window of variable length in the genome of *E. coli* W. Points represent probability values for different length values.

**
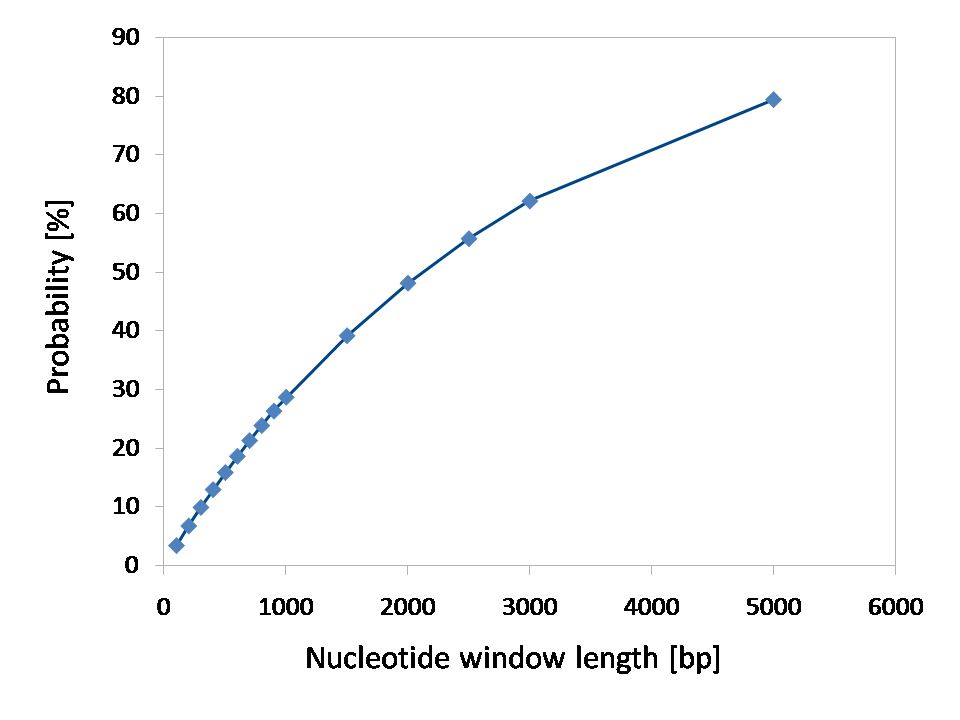
**

**Table S1.** Primers used in this study.

| PAtail_F (*Eco*RI)^a^ | CTTCGAATTCGCGGCCGCTTCTAGAGGAATGTTTTGATCAAACAGAGGGC |
| --- | --- |
| PAtail_R (*Pst*I) | CTTCCTGCAGCGGCCGCTACTAGTATGCCCGAACGAACTGGTTTA |
| PBtail_F (*Eco*RI) | CTTCGAATTCGCGGCCGCTTCTAGAGCATCAACAACTATGCTTAGTGTAG |
| PBtail_R (*Pst*I) | CTTCCTGCAGCGGCCGCTACTAGTACATCGCTTACGGTCAATTGTTGAC |
| VF2 | TGCCACCTGACGTCTAAGAA |
| VR | ATTACCGCCTTTGAGTGAGC |
| PA_F | TTACACATCCCGCCATCAGC |
| PB_R | GCAATTTCGCCAGACAAGCA |
| P1_F | TAGCCAGTCTGAATGACCTGTCAC |
| P2_R | CCTCAGATCCTTCCGTATTTAGCC |
| P3_F | CAAACAGCGTTTGCGACATCCT |
| P4_F | GCCCGACTGATACGTTGATTTTCC |
| P5_R | AAGGCTTAAGTAGCACCCTCGCAA |

a) The restriction sites used for cloning are reported in brackets and their sequence is underlined.
